# A Dual-Purpose Approach for an Anti-Emetic NK1R Antagonist as a Chemosensitizer and Cardioprotectant in a Preclinical Model of Triple-Negative Breast Cancer

**DOI:** 10.3390/ijms26199353

**Published:** 2025-09-25

**Authors:** Miguel Muñoz, Rafael Coveñas, Younus Zuberi, Zara Italia, Tan Hoang, Zal Italia, Prema Robinson

**Affiliations:** 1 Research Laboratory on Neuropeptides (IBIS), Virgen del Rocío University Hospital, 41013 Sevilla, Spain; miguel.mmunoz@gmail.com; 2 Laboratory of Neuroanatomy of the Peptidergic Systems, Institute of Neuroscience of Castilla and León (INCYL), University of Salamanca, 37007 Salamanca, Spain; covenas@usal.es; 3 Group GIR USAL: BMD (Bases Moleculares del Desarrollo), University of Salamanca, 37007 Salamanca, Spain; 4 Department of Infectious Diseases, Infection Control and Employee Health, The University of Texas MD Anderson Cancer Center, Houston, TX 77030, USA; younus.a.zuberi@uth.tmc.edu (Y.Z.); italia.zara@yahoo.com (Z.I.); tmhoang@mdanderson.org (T.H.); zsitalia1@mdanderson.org (Z.I.)

**Keywords:** triple-negative breast cancer (TNBC), cardiotoxicity, substance P, neurokinin-1 receptor, doxorubicin, aprepitant

## Abstract

Doxorubicin (Dox) is considered one of the most effective treatments for triple-negative breast cancer (TNBC); however, it can cause limited efficacy, recurrence/chemoresistance, and cardiotoxicity. Using a murine preclinical MDA-MB-231 TNBC model, we determined that targeting the substance P/neurokinin-1 receptor signaling axis can increase efficacy of the standard-of-care treatment currently used for TNBC, i.e., doxorubicin (Dox), while also attenuating Dox-induced, cardiotoxicity in TNBC. The in vivo studies outlined in this manuscript validate aprepitant (AP), a neurokinin-1 receptor antagonist, as a safe, dual-purpose chemosensitizer and cardioprotectant. These studies provide preclinical evidence supporting further evaluation of a continuous daily AP regimen in TNBC models in combination with Dox, laying the groundwork for future investigations into its safety, dosing, and potential clinical application. Because AP is already FDA-approved for single-dose anti-emetic use, repurposing it for chronic administration offers a rapid path to clinical translation, with the potential to redefine chemotherapy paradigms and tangibly improve survival and quality of life in TNBC.

## 1. Introduction

Every year, nearly 2.3 million breast cancer cases are diagnosed globally, and approximately 15–20% belong to the triple-negative (TNBC) subtype [1,2]. TNBC tends to occur more frequently in younger women (often <50 years old), African-American/non-Hispanic Black women (approx. 27–28 per 100,000 vs. ~13 per 100,000 in non-Hispanic White women) and women with BRCA1 mutations [1,2].

TNBC is characterized by aggressive clinical characteristics, early peak of distant recurrences at 3 years after diagnosis, and a high mortality rate within the first 5 years [3,4]. Due to the lack of estrogen receptor/progesterone receptor/human epidermal growth factor receptor 2, TNBCs do not respond to hormone-based therapies; hence, chemotherapy still remains the standard-of-care treatment. Doxorubicin (Dox), an anthracycline-class medication is considered one of the most effective treatments for TNBC [5,6]. It can cause limited efficacy, recurrence/chemoresistance, and cardiotoxicity [7,8,9]. Novel combinations are urgently needed to increase the efficacy of Dox and prevent cardiotoxicity and chemoresistance.

Substance P (SP) is a peptide involved in pain transmission, but after binding to the neurokinin-1 receptor (NK1R), it also promotes tumor cell proliferation, inhibits apoptosis, increases angiogenesis, and enhances migration and metastasis [10,11,12]. Our group demonstrated elevated NK1R expression in TNBC cells relative to normal mammary epithelial cells [13].

Moreover, emerging evidence from our in vitro studies with TNBC cell lines [14,15] and cardiotoxicity studies in immunocompetent mice without cancer [16] indicates that the SP/NK1R signaling axis is involved in decreased sensitivity to chemotherapeutic agents and the induction of side effects. However, so far, there are no in vivo studies that have investigated whether targeting SP/NK1R can be beneficial in vivo to increase the efficacy of the standard-of-care treatment currently used for TNBC, i.e., doxorubicin (Dox), while also preventing Dox-induced cardiotoxicity in the same immunocompromised nude mice with TNBC. In the current studies, we determined if a routinely used FDA-approved anti-emetic NK1R antagonist, aprepitant (AP), can possibly be repurposed to increase the efficacy of Dox in TNBC and at the same time prevent the cardiotoxic side effects of doxorubicin.

Aprepitant is a highly selective NK1R antagonist that is approved for the prevention of chemotherapy-induced nausea and vomiting (CINV) in single-dose regimens. By blocking the binding of substance P (SP) to NK1R, AP interferes with a signaling axis that plays a critical role in cancer progression. In triple-negative breast cancer (TNBC) and other malignancies, SP/NK1R signaling promotes tumor cell proliferation, migration, invasion, and survival, while also stimulating angiogenesis and supporting the Warburg effect to create a favorable metabolic environment for tumor growth. By antagonizing NK1R, AP can inhibit these pro-tumorigenic pathways, potentially sensitizing cancer cells to chemotherapy while reducing tumor progression. Importantly, AP is orally bioavailable, well-tolerated at therapeutic doses, and primarily metabolized by CYP3A4, making it an attractive candidate for repurposing as a mechanism-based, dual-function agent that enhances chemotherapy efficacy and mitigates side effects such as cardiotoxicity.

The studies outlined underline the crucial role played by the SP/NK-1R system in TNBC development, providing a novel treatment strategy that can be enormously beneficial for breast cancer patients.

## 2. Results

### 2.1. NK1 Receptor Antagonism Enhances the Antitumor Efficacy of Dox

We determined whether NK1R antagonism enhances the antitumor efficacy of Dox. First, we injected 2 × 10^6^ MDA-MB-231 TNBC cells into the cleared mammary pads of 3–4-week-old female athymic *nu/nu* mice. Starting when tumors reached an average size of ~40–60 mm^3^, mice were randomized to receive AP (10 mg/kg) oral gavage, 5 days/week (M-F) along with Dox (4.5 mg/kg) or Dox (2.25 mg/kg) intravenously every week for four administrations. At 28 days post-treatment of the first dose of Dox/AP, the mice were euthanized.

**Figure 1:**
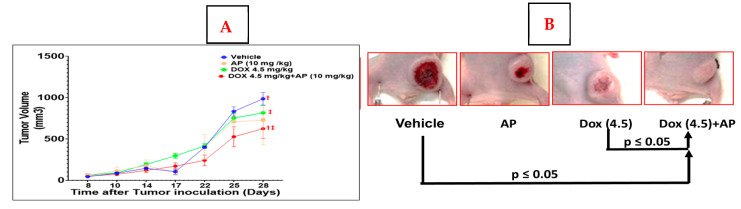
Addition of AP to Dox regime led to a better outcome of TNBC in a murine preclinical cell line xenograft model. (**A**) We determined that there was a significant decrease in tumor volume in the Dox (4.5 mg/kg) + AP (10 mg/kg) versus the vehicle and the Dox alone (4.5 mg/kg) groups (*p* ≤ 0.05, ANOVA/Tukey’s test; *n* = 4–6). Although AP alone caused a decrease in tumor volume compared to the vehicle group, the difference was not significant. (**B**) Representative tumor after each treatment, showing Dox + AP resulted in the smallest tumors.

We determined that there was a significant decrease in tumor volumes in the Dox (4.5 mg/kg) + AP (10 mg/kg) versus the vehicle and the Dox alone (4.5 mg/kg) groups (*p* ≤ 0.05, two-way ANOVA/Tukey’s test). Although AP alone caused a decrease in tumor volume compared to the vehicle group, the difference was not significant. There were no significant differences betwen the Dox (2.5 mg/kg) with and without AP. Compared to the Dox (4.5mg/kg) alone and the vehicle groups, the mean tumor volume in the Dox (4.5 mg/kg) + AP group at the end of experiment was significantly decreased (Figure 1A,B; Dox (4.5 mg/kg); 816 ± 220 mm^3^, *n* = 6 vs. Dox (4.5 mg/kg) + AP (10 mg/kg); 623 ± 291 mm^3^, *n* = 6 (‡; *p* = 0.0148, two-way ANOVA with Tukey’s post hoc test); vehicle; 985 ± 167 mm^3^; *n* = 5 vs. Dox (4.5 mg/kg) + AP (10 mg/kg); 623 ± 291 mm^3^, *n* = 6 *(*†; *p* = 0.0321, two-way ANOVA with Tukey’s post hoc test).

### 2.2. Combination of NK1 Receptor Antagonist with Dox Leads to Significantly Greater Decrease in Levels of the Proliferation Marker, Ki-67, Compared to Either Agent Alone

**Figure 2:**
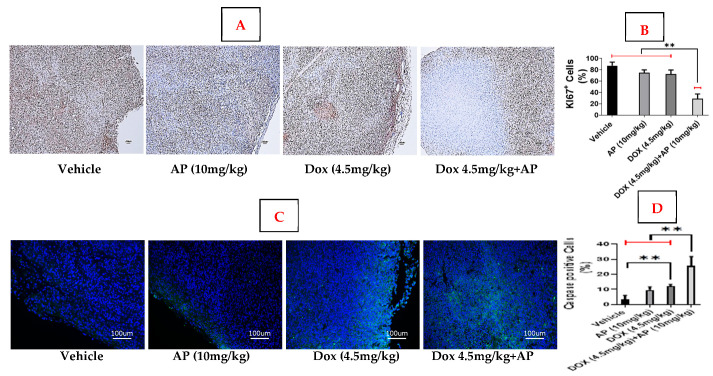
Levels of KI-67 and cleaved caspase-3 in the murine preclinical MDA-MB 231 model. (**A**) Ki-67 index reported as % of positive cells (brown-stained within nucleus) (at least 1000) by IHC in vehicle, AP (10 mg/kg), Dox (4.5 mg/kg), Dox (4.5 mg/kg) + AP (10 mg/kg). Magnification 40×. (**B**) Significant decrease in Ki-67 index seen in the Dox (4.5 mg/kg) + AP (10 mg/kg) vs. the vehicle and the Dox alone (4.5 mg/kg) group (**, *p* ≤ 0.01, ANOVA/Tukey’s; *n* = 2–3). (**C**) Cleaved caspase-3 levels reported as % of positive cells (green fluorescent-stained within nucleus (nucleus; blue DAB-stained); (at least 1000); in vehicle, AP (10 mg/kg), Dox (4.5 mg/kg), and Dox (4.5 mg/kg) + AP (10 mg/kg) groups. Magnification 200×. (**D**) Significant decrease in cleaved caspase-3 seen in the Dox (4.5 mg/kg) + AP (10 mg/kg) vs. all other groups, and between vehicle and the Dox alone (4.5 mg/kg) groups (**, *p* ≤ 0.01, one-way ANOVA; *n* = 2–3).

Other studies have shown that in TNBC patients, the most relevant cutoff value for Ki-67 (the proliferation marker) for prognosis is 30% (*p* = 0.008). At the cutoff point of 30%, worse disease-free survival (DFS) and overall survival (OS) were observed in the Ki-67^highK^ group [17]. We determined that the Ki-67 index, reported as the percentage of nuclear staining-positive cells (at least 1000), was 87.33% ± 6.42 in the untreated vehicle group, 75% ± 5 in the AP alone group, and 72.33% ± 7.5 in the Dox alone group. The Ki-67 index was significantly lower in the Dox (4.5 mg/kg) + AP group (29% ± 8.48) vs. the Dox (4.5 mg/kg) and vehicle groups (*p* ≤ 0.01, one-way ANOVA with Tukey’s post hoc test; *n* = 2–3); see Figure 2A,B.

### 2.3. Combination of NK1 Receptor Antagonist with Dox Leads to Significantly Greater Increase in Levels of the Apoptosis Marker, Cleaved Caspase-3, Compared to Either Agent Alone

We determined whether AP had any effect on Dox-induced apoptosis, based on the levels of cells positive for the apoptotic marker cleaved caspase-3 in the murine preclinical MDA-MB 231 model. We determined that the % of cells positive for the apoptotic marker, cleaved caspase-3 positive cells, was 3.33% ± 2.8 in the untreated vehicle group, 9.67% ± 2.12 in the AP group, and 12% ± 1.4 Dox group. The level of cleaved caspase-3 was significantly higher in the Dox+AP group (25.5% ± 6.3) vs. all other groups (*p* < 0.01; one-way ANOVA; *n* = 2–3). There was also significantly more cleaved caspase-3-positive cells in the Dox versus the vehicle group; *p* < 0.01; one-way ANOVA; *n* = 2–3); see Figure 2C,D.

### 2.4. NK1 Receptor Antagonism Protects Against Dox-Induced Cardiotoxicity

**Figure 3:**
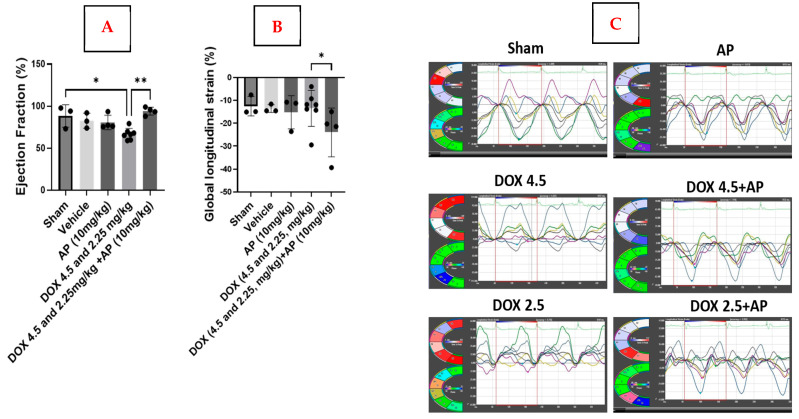
Addition of AP to Dox regime protects against DOX-induced cardiotoxicity in the murine preclinical cell line xenograft model. (**A**) Significant increase in ejection fraction seen in the Dox (4.5 mg/kg and 2.25 mg/kg) + AP (10 mg/kg) groups versus the vehicle and the Dox alone (4.5 mg/kg and 2.25 mg/kg) groups (*, *p* ≤ 0.05, **, *p* ≤ 0.01; Kruskal–Wallis/Dunn’s test; *n* = 3–7). (**B**) Significant decrease in global longitudinal strain was seen in the Dox (4.5 mg/kg and 2.25 mg/kg) + AP (10 mg/kg) groups versus the Dox alone (4.5 mg/kg and 2.25 mg/kg) groups (*p* ≤ 0.05, Kruskal–Wallis/Dunn’s test; *n* = 3–7). (**C**) Global longitudinal strain images representing the sham, AP (10 mg/kg), Dox (4.5 mg/kg), Dox (4.5 mg/kg) + AP (10 mg/kg), Dox (2.25 mg/kg), and Dox (2.25 mg/kg) + AP (10 mg/kg) groups.

We determined the effects of AP on Dox-induced cardiotoxicity. We firstly determined that there was a significant decrease in the ejection fraction (EF) in response to Dox (4.5 mg/kg and 2.25 mg/kg) versus the sham (sham; 87.64 ± 9.6%, *n* = 3 vs. Dox (4.5 mg/kg and 2.25 mg/kg); 66.88 ± 6.74%, *n* = 7; *, *p* = 0.0120, Kruskal–Wallis with Dunn’s post hoc test). Most importantly, the addition of AP to the Dox regime led to a significant increase in the mean EF compared to the Dox group without AP (Figure 3A; Dox (4.5 mg/kg and 2.25 mg/kg) + AP (10 mg/kg); 94.1 ± 4.6%, *n* = 4 vs. Dox (4.5 mg/kg and 2.25 mg/kg); 66.88 ± 6.74%, *n* = 7; **, *p* = 0.0006, Kruskal–Wallis with Dunn’s post hoc test). Other parameters, such as fractional shortening, did not show significant differences between the different groups. We also performed Global Longitudinal Strain (GLS) analysis using speckle tracking echocardiography to quantify the extent to which the heart muscle shortened during left ventricular (LV) systolic contraction. GLS is more sensitive than the routinely used ejection fraction determinant and is an early indicator of myocardial dysfunction, specifically in patients that are being treated with cardiotoxic chemotherapeutic agents, such as Dox. GLS evaluates changes in length relative to the initial length (strain = final length [L]/initial length [L0]). We determined that GLS was significantly reduced upon the addition of AP to the Dox regime compared to the Dox group without AP (Figure 3B,C; Dox (4.5 mg/kg and 2.25 mg/kg) + AP (10 mg/kg); −24 ± 10.6%, *n* = 4 vs. Dox (4.5 mg/kg and 2.25 mg/kg); −13.49 ± 7.8%, *n* = 6; *, *p* = 0.0203, Kruskal–Wallis with Dunn’s post hoc test). There was no difference in radial strain between the different groups.

## 3. Discussion

In the current study, we determined that a routinely used FDA-approved anti-emetic NK1R antagonist, AP, can possibly be repurposed to increase the efficacy of Dox in TNBC and at the same time prevent cardiotoxic side effects of doxorubicin. The SP/NK-1R system has been shown to play a derogatory role in cancer based on the following key roles of this system: (1) NK-1R is overexpressed by cancer cells [18,19,20]; (2) SP, a peptide, is produced by tumor cells, and immune cells located in the tumor microenvironment, and released from nerve terminals [18,19,20]; (3) SP, via interaction with its high-affinity NK1R, which is overexpressed on cancer cells, leads to mitogenesis of cancer cells [18,19,20]; (4) antagonism of NK-1R with antagonists such as AP and others prevents SP/NK1R interaction, leading to antitumor effects. SP/NK1R interaction plays a derogatory role in cancer through many mechanisms; this interaction is known to be beneficial for the survival of tumor cells, as well as for tumor development. The interaction of SP with NK1R on cancer cells is known to induce proliferation, migration, and antiapoptosis of cancer cells [18,19,20]. Furthermore, this interaction is known to stimulate the survival and progression of cancer by stimulating the angiogenesis/development of new blood vessels, thereby providing an additional supply of nutrients to the cancer cells, and the Warburg effect, creating a unique metabolic environment within tumors wherein cancer cells preferentially use anaerobic glycolysis to produce energy, even in the presence of oxygen.

Importantly, in addition to neuronal and immune cell-derived SP, circulating SP can reach cancer cells from the bloodstream [20]. A case–control study on 41 women with breast cancer and 34 healthy controls determined that the serum SP values of the patients showed significantly higher levels than those of the healthy individuals [21]. Also determined in the study was that increased NK1R tissue distribution was observed in patients with breast cancer compared with the controls. These studies highlighted the involvement of the SP/NK1R complex in breast cancer incidence [21].

Moreover, the involvement of the SP/NK1R complex in breast cancer pathogenesis was highlighted in a recent study published in 2025 by Guttierez et al. The study used MDA-MB-231, SP stimulation, and 3D cell culture models to better reproduce the heterogenous microenvironment of solid tumors observed in vivo. They determined that the TNBC cell line was susceptible to SP stimulation, and most importantly, NK1R antagonists (AP) attenuated SP-induced proliferation [22].

A recent publication by Padmanaban et al. using a TNBC murine breast cancer cell line, 4T1, and two Patient-Derived Xenografts (PDX’s), determined that primary tumor growth was significantly reduced by AP [23]. However, the study did not use the human TNBC cell line, and AP was delivered intratumorally in the 4T1 model while it was delivered intraperitoneally in the PDX model. In our study, we did not see a significant reduction in tumor growth between the AP and vehicle group; this could possibly be because we delivered AP by oral gavage instead of intratumorally or intraperitoneally. We utilized the oral administration method since our long-term goal is to administer AP in patients, we therefore picked a mode of administration that is amenable to the clinical setting, and which is currently being used to prevent chemotherapy-induced nausea. We chose the dose of AP based on the following: the dose of AP used in humans before the start of chemotherapy is 95 mg/day; the average weight of an adult proposed in pharmacology studies is 60 kg. Therefore, the dose of AP used in humans is 95/60, i.e., approx. 1.583 mg/kg. Based on this, the HED to be used in mice is 1.583 mg/kg × 12.3 = approx. 19.475 mg/kg. Since we applied a combinational strategy, we used a dose equivalent to half of the HED (10mg/kg).

However, in two osteosarcoma and hepatoblastoma xenograft models, aprepitant oral solution was administered at doses higher than those used in this study at 80 mg/kg/day. Tumor weight and size at the end of the experiment decreased significantly in the treatment group [12,24]. Therefore, the lack of reduction in tumor growth by AP alone in our study is probably related to the dose used in our oral administration regime; at higher oral doses, the antitumor effect will probably be more evident. Although other studies have utilized doses of Dox as high as 9 mg/kg [25], in our experiments, utilizing this high dose led to the death of all tumor-bearing nude mice within the first week; therefore, we opted to use a lower dose of Dox. In addition, our previously published cardiotoxicity model in cancer-free immunocompetent mice utilized a lower dose of 4.5 mg/kg of Dox [16]. Most importantly, since we were studying the effects of a combination regime of AP and Dox, we elected to utilize lower combination doses of each agent to avoid toxicity and untimely deaths, specifically those associated with Dox. In this study, the results show that AP alone did not reduce the tumor size significantly with the dose used, while in combination with Dox, it did. The potential reasons behind this potentiation effect could be based on the pharmacokinetics of the combination. Doxorubicin is a major substrate of CYP3A4. Therefore, the co-administration of Dox with CYP3A4 inhibitors is expected to increase systemic Dox exposure. Thus, a possible explanation for the potentiating effect of AP could be due to its ability as a CYP3A4 inhibitor to potentially increase the area under the curve (AUC) of Dox. This could be the reason for the greater anticancer effects of Dox.

Adding credence to our current study is a study by a group in Norway [26], wherein, 13,811 women who were diagnosed with early breast cancer in Norway and administered chemotherapy and anti-emetics were studied. The association between AP use and distant disease-free survival (DDFS) and breast cancer-specific survival (BCSS) was evaluated. This important study demonstrated that the administration of AP during chemotherapy treatment led to a better prognosis for women with non-luminal early breast cancer, particularly TNBC.

We do not know the mechanisms by which the combination of AP and Dox potentiated the cytotoxic activity while it attenuated cardiotoxic activity. There could be mutually exclusive pathways, with some present only in cardiomyocytes, such as Topoisomerase 2b, an enzyme that induces Dox-induced double-strand DNA breaks and subsequent DNA damage, and those present only in tumors, such as the Raf/Mek/Erk, Notch1, PI3K/AKT/Mtor pathways, which are known to mediate breast cancer progression and resistance to chemotherapy. These pathways may be respectively involved in the induction of SP/NK1R-mediated cardiotoxicity and decreased anti-tumor efficacy in response to Dox. Studies are underway in our laboratory to pinpoint the exact mechanisms involved in these dual opposing roles of SP/NK1R interaction in Dox-mediated cardiotoxicity and the induction of decreased efficacy of Dox.

Importantly, AP has been shown to be safe and well tolerated in humans. We have previously shown that a patient with obstructive pulmonary disease (COPD) and non-small-cell lung carcinoma (NSCLC) received 45 days of aprepitant (compassionate use, 1140 mg/day) and radiotherapy. After 6 months of treatment, the tumor mass disappeared and no side effects were observed [27]. Other clinical investigators showed that administration of 80 mg/day of AP (initially for seven months), followed by an increase to 120 mg every third day was well-tolerated and effective to attenuate CA153 tumor marker levels and emesis in a metastatic breast cancer patient with nausea and vomiting, who was unresponsive to all standard antiemetic therapy [28].

The in vivo studies outlined in this manuscript validate AP as a safe, dual-purpose chemosensitizer and cardioprotectant. These studies set the stage for supporting an IND and Phase I trial of using a continuous daily AP regime in TNBC patients undergoing Dox treatment. Because AP is already FDA-approved for single-dose anti-emetic use, repurposing it for chronic administration offers a rapid path to clinical translation, with the potential to redefine chemotherapy paradigms and tangibly improve survival and quality of life in TNBC. Notably, repurposing AP to achieve dual antitumor and cardioprotective effects could be potentially achievable only under continuous daily AP administration, a marked deviation from the single-dose, antiemetic use for which the drug is currently approved. This novel dosing strategy is central to our therapeutic concept. By shifting the paradigm from single-use to sustained exposure, we aim to repurpose the safe drug AP as a mechanism-based, dual-function agent enhancing Dox efficacy while simultaneously reducing its cardiotoxicity. This strategy directly addresses two of the most pressing challenges in TNBC treatment: chemoresistance and treatment-limiting cardiotoxicity.

## 4. Material and Methods

### 4.1. TNBC Xenograft Model

We injected MDA-MB-231 TNBC cells into the cleared mammary pads of 3–4-week-old female athymic *nu/nu* mice (Stock no. 002019, Jackson labs, Bar Harbor, ME, USA). Tumor volume [*V* = 0.5 × (*L* × *W*^2^)] was measured by calipers. Starting when tumors were approximately an average size of ~40–60 mm^3^, mice were randomized to receive AP (Catalog No.S1189; Selleckchem, Houston, TX, USA; 10 mg/kg, oral gavage, 5 days/week (M-F) with/without Dox (Catalog No. Catalog No.S1208; Selleckchem, Houston, TX, USA). The doses of AP used for these preliminary studies were as per other tumor efficacy studies [12]. We used the following doses of Dox (4.5 mg/kg or 2.25 mg/kg) intravenously every week for 4 administrations. At 28 days post-treatment of the first Dox/AP dose, the mice were euthanized. There were 6 groups of 4–7 mice as follows: (1) vehicle; (2) AP (10 mg/kg); (3) Dox (4.5 mg/kg); (4) Dox (4.5 mg/kg) +AP (10 mg/kg); (5) Dox (2.25 mg/kg); (6) Dox (2.25 mg/kg) + AP (10 mg/kg).

### 4.2. ECHO Procedure

We used the MD Anderson Small Animal Imaging Facility research core to perform echo and strain analyses on all 6 groups of mice stated above, including both MDA-MB-231 tumor-bearing mice and sham (non-tumor-bearing) mice. A Vevo 2100 ultrasound machine that was equipped with a 30 MHz transducer (Visualsonics, Toronto, ON, Canada) was used to measure cardiac function in mice from the above 6 groups. Standard B-mode (2D) and M-mode images were taken of each animal. Visualsonics VevoStrain software was used to analyze the data from the M-mode images. Results are expressed as the mean values of each echo readout ± Standard Error of Mean (SEM) for each group.

### 4.3. Strain Analyses

We used parasternal long-axis B-mode loops to measure strain using the VisualSonics Vevo 2100 system (VisualSonics, Toronto, ON, USA; https://www.visualsonics.com/product/software/vevo-lab; accessed on 23 September 2025). B-mode loops were acquired and then imported into Vevo Strain software. Three consecutive cardiac cycles were selected, and the endocardial border was traced. Following adequate endocardial delineation, the epicardial border was subsequently traced. Both radial and longitudinal strain were determined. Radial strain is defined as the percentage change in myocardial wall thickness during the cardiac cycle. The measurement indicates myocardial deformation directed inward toward the center of the left ventricular (LV) cavity, representing radial myocardial function. Longitudinal strain (LS) measures the percentage change in the length of the left ventricle from base to apex, typically assessed from the endocardial surface in the long-axis view. This reflects myocardial deformation along the longitudinal axis, with negative values indicating shortening. Results are expressed as the mean percentage ± Standard Error of Mean (SEM) for each group.

### 4.4. Ki-67 Immunohistochemical Staining

Briefly, formalin-fixed paraffin-embedded tumor blocks were sectioned at 5 microns. The sections were deparaffinized by two treatments with xylene (10 min for each treatment), followed by treatment in 100% ethanol (5 min, 2 times), 90% ethanol (5 min, 2 times), 80% ethanol (5 min, 2 times), and 70% ethanol (5 min). The slides were then washed twice with 1XPBS (5 min each wash) and then treated with citrate phosphate buffer at pH6.0 for antigen retrieval. The slides were then washed twice with 1XPBS, and then incubated for 10 min in Ki67 (Cat#9027, Cell Signaling Technology, Beverly, MA, USA), and developed using an automated bond polymer refine detection kit (Cat #DS 9800, Leica Biosystems, Buffalo Grove, IL, USA), counterstained with hematoxylin, dehydrated, and cover-slipped. The brown DAB cleaved KI-67 positive stained cells (around the tumor periphery) were counted at 100× magnification within a total of 1000 cells. Ki-67 at the tumor’s periphery reflects the proliferation rate of cancer cells at the tumor’s edges, which is a crucial factor in determining breast cancer aggressiveness and risk of recurrence. A higher Ki-67 index, especially in the periphery, generally indicates a more aggressive tumor and is used by pathologists to help classify breast cancer, inform treatment decisions, and predict the likelihood of relapse. We therefore opted to determine the levels of KI67 specifically in 1000 cells around the periphery.

### 4.5. Cleaved Caspase-3 Immunofluorescence Staining

Slides were sectioned at 5 microns from the formalin-fixed paraffin-embedded blocks. The sections were deparaffinized by two treatments with xylene (10 min for each treatment), followed by treatment in 100% ethanol (5 min, 2 times), 90% ethanol (5 min, 2 times), 80% ethanol (5 min, 2 times), and 70% ethanol (5 min). The sections were then dehydrated in 50% ethanol (5 min), 70% ethanol (5 min), and 100% ethanol (5 min) at room temperature. The slides were air-dried, and using the hydrophobic pen (ImmEdge PAP pen, Vector Labs), boundaries were drawn around each tissue section. The sections were then (a) blocked using blocking buffer containing 10% donkey serum, 0.3% Triton -X 100 in 0.1 M phosphate buffer (PB) for 1 h at room temperature, (b) incubated with primary antibody; cleaved caspase-3 (Cat#9661; Cell Signaling Technology, Beverly, MA, USA) diluted in blocking buffer overnight at 4 °C, and (c) washed once with 0.1 M PBS, followed by incubation with secondary antibodies, anti-rabbit IgG (Cat # 4412; Cell Signaling Technology, Beverly, MA, USA), for 1 h at room temperature. The sections were washed with 0.1 M PBS to get rid of unbound secondary antibody, air-dried, and cover-slipped with Prolong Gold Antifade reagent (Cat#8961; Cell Signaling Technology, Beverly, MA, USA). The sections were imaged using an Olympus FV3000 confocal microscope at 200X magnification. The green, fluorescent cleaved caspase-3 positive cells were counted within a total of 1000 cells.

#### Statistical Analyses

Data were first assessed for normality using the Shapiro–Wilk test to determine the appropriate statistical approach. For datasets that were normally distributed, differences between multiple groups were evaluated using one-way analysis of variance (ANOVA), which allows for comparison of means across three or more groups while controlling for type I error. To identify specific group differences following ANOVA, pairwise comparisons were performed using Tukey’s post hoc test, which adjusts for multiple comparisons. For datasets that did not meet the assumptions of normality, non-parametric tests were employed. The Kruskal–Wallis test was used to evaluate differences between multiple groups, as it does not assume a normal distribution and is robust for skewed or ordinal data. Following a significant Kruskal–Wallis result, pairwise comparisons were conducted using Dunn’s test with appropriate correction for multiple testing. A *p*-value of <0.05 was considered statistically significant for all analyses. All statistical analyses were conducted using GraphPad Prism 7.03 (San Diego, CA, USA).

## 5. Conclusions

Our study demonstrates that the NK1R antagonist aprepitant (AP) has significant potential as a dual-function agent in triple-negative breast cancer (TNBC) therapy, simultaneously enhancing doxorubicin (Dox) efficacy and mitigating its cardiotoxic effects. By targeting the substance P/neurokinin-1 receptor (SP/NK1R) axis, which promotes TNBC proliferation, survival, migration, and metabolic adaptation, AP offers a mechanism-based approach to overcoming chemoresistance. Importantly, continuous daily administration of AP—distinct from its conventional single-dose antiemetic use—represents a novel and cliniclly translatable strategy to maximize therapeutic benefit.

These findings have several practical implications. First, AP could be repurposed to improve the efficacy and safety of standard chemotherapy regimens for TNBC, potentially reducing treatment-related cardiotoxicity and enhancing patient outcomes. Second, this approach underscores the value of targeting the SP/NK1R signaling pathway as a complementary strategy alongside cytotoxic therapy.

Future studies should focus on optimizing AP dosing and administration schedules, evaluating long-term safety in non-tumor and tumor-bearing models, and exploring its effects across different TNBC subtypes. Additionally, clinical trials are warranted to assess AP’s dual chemosensitizing and cardioprotective roles in TNBC patients. Collectively, our work provides a strong preclinical foundation for translational research, offering a pathway toward improving the survival and quality of life in patients with TNBC while advancing the broader understanding of mechanism-based cancer therapies.

## Data Availability

Data is contained within the article.
